# Symptom improvement with dosing above 9 g of low-sodium oxybate: effectiveness and safety in participants with narcolepsy in the DUET study

**DOI:** 10.1007/s44470-026-00193-6

**Published:** 2026-10-15

**Authors:** Jerald H. Simmons, Logan D. Schneider, Chad M. Ruoff, David T. Plante, Deborah A. Nichols, Teresa L. Steininger, Marisa Whalen, M. Todd Kirby, Jing Dai, Alyssa Cairns, Richard K. Bogan

**Affiliations:** 1 Comprehensive Sleep Medicine Associates, Houston, TX USA; 2 Sleep Education Consortium, Sugar Land, TX USA; 3 Stanford University Center for Sleep Sciences and Medicine, Stanford University, Stanford, CA USA; 4 Mayo Clinic, Scottsdale, AZ USA; 5 Department of Psychiatry, University of Wisconsin–Madison, Madison, WI USA; 6 Jazz Pharmaceuticals, Palo Alto, CA USA; 7 Jazz Pharmaceuticals, Philadelphia, PA USA; 8 Department of Psychiatry & Health Behavior, Medical College of Georgia at Augusta University, Augusta, GA USA; 9 University of South Carolina School of Medicine, Columbia, SC USA; 10 REST Technology, Inc.,, Sugar Land, USA TX

**Keywords:** Low-sodium oxybate, Narcolepsy, Oxybate titration, Patient-centric study, Patient-reported outcome, Polysomnography, Clinical decision-making, Drug utilization, Off-label use, Patient-centered care

## Abstract

**Purpose:**

Evaluate effectiveness/safety of low-sodium oxybate (LXB; Xywav^®^) dosages > 9 g/night in participants with narcolepsy (type 1 [NT1] or 2 [NT2]).

**Methods:**

Jazz DUET (prospective, multiple-cohort, open-label study [NCT05875974]) included screening, 9-day baseline (LXB 9 g/night [“LXB 9 g BL”]), 2–8-week titration, 2-week stable-dose, 9-day end-of-treatment (LXB > 9 g– ≤ 12 g [“optimized LXB > 9 g EOT”]; twice-nightly, first/second max doses = 7.5/4.5 g), and 2-week follow-up. Endpoints included Epworth Sleepiness Scale (ESS), Narcolepsy Severity Scale–clinical trials version (NSS-CT), Patient Global Impression of Severity (PGI-S) and Change (PGI-C), and polysomnography (PSG)-measured respiratory assessments.

**Results:**

Forty-eight participants took LXB > 9 g/night (mean ± SD age: 39.2 ± 10.9 years; 62.5% female); 44 (NT1, *n* = 28; NT2, *n* = 16) completed the study. Mean ± SD stable-LXB dosage (*n* = 45) was 11.1 ± 1.0 g/night (most common [43.8%], 12 g/night). From LXB 9 g BL to optimized LXB > 9 g EOT, mean ± SE ESS scores (11.4 ± 0.8 to 8.3 ± 0.9), NSS-CT (NT1) scores (17.3 ± 2.2 to 9.4 ± 2.1), and NSS-2-CT (NT2) scores (9.7 ± 1.7 to 4.3 ± 1.6) improved. Fewer participants rated their disease as severe (61.4% to 29.5%), and most (92.3%) reported their disease improved. AHI (1.8 ± 0.4 to 1.6 ± 0.3), CAI (0.01 ± 0.01 to 0.01 ± 0.00), and mean SpO_2_ (95.8 ± 0.2 to 95.8 ± 0.1) remained stable. Common TEAEs included nausea (12.5%), vomiting (12.5%), and headache (10.4%).

**Conclusion:**

Participants taking LXB > 9 g/night experienced further improvements in narcolepsy symptoms versus 9 g/night, suggesting some patients may require higher dosages for optimal benefit. No changes in mean respiratory PSG measures were observed. TEAEs align with the known safety profile. Findings support individualized clinical decision-making with higher LXB dosages.

**Clinical trial registration:**

Trial name: A Study to Investigate the Effect of XYWAV on Sleepiness, Polysomnography, and Functional Outcomes in Participants With Idiopathic Hypersomnia or Narcolepsy; URL: https://clinicaltrials.gov/study/NCT05875974; ID number: NCT05875974.

**Brief summary:**

**Current knowledge/study rationale:**

Narcolepsy is a heterogeneous disorder requiring individualized treatment strategies to optimize symptom management. This study fills a critical knowledge gap by evaluating effectiveness and safety of low-sodium oxybate (LXB) dosing regimens higher (> 9–12 g/night) than the recommended dosage (6–9 g/night).

**Study impact:**

Participants with narcolepsy taking LXB > 9 g/night experienced further overall symptom benefit compared with 9 g/night at baseline, suggesting some patients may require higher dosages to achieve optimal therapeutic benefit. The safety profile, including respiratory safety parameters, was comparable to that observed at 9 g/night. Findings demonstrate that LXB can be utilized at dosages up to 12 g/night to optimize effectiveness, supporting individualized clinical decision-making.

**Plain language summary:**

Low-sodium oxybate (LXB; Xywav^®^) is a medication used to treat narcolepsy symptoms. The recommended dosage range is 6 g to 9 g/night, but it has been reported that some people take higher dosages (greater than 9 g/night). This study was the first to evaluate higher dosages of LXB (greater than 9 g/night and up to 12 g/night). Before this study, these higher dosages had not been formally studied. This study was done to evaluate whether higher dosages improve narcolepsy symptoms and/or impact side effects. LXB is a central nervous system depressant, so there was a need to see if higher dosages could impact breathing during sleep. The study enrolled people who were already taking LXB at 9 g/night (the highest recommended dose) and who clinicians thought could benefit from higher dosages. While taking LXB 9 g/night, participants completed tests to measure their symptoms. Participants then worked with a clinician for 2 to 8 weeks to find a higher LXB dosage that worked best for them. The highest dosage allowed was 12 g/night. By the end of the study, the average dosage taken was 11.1 g/night, and almost half (44%) of participants were taking 12 g/night. Once participants found their ideal dosage, they stayed on this dosage for 2 weeks. Then, they repeated the same tests that they completed before. Forty-eight participants started the study. Forty-four completed the study. With higher LXB dosages (compared to 9 g/night), participants on average had less excessive daytime sleepiness, fewer cataplexy attacks, and less severe narcolepsy symptoms. At the beginning of the study when taking LXB 9 g/night, most participants (61.4%) said their disease was severe. After taking higher dosages, this dropped to 29.5%. At the end of the study, nearly all (92.3%) said their overall disease improved with higher dosages. Nobody said their disease worsened. In general, higher dosages did not worsen breathing during sleep. The most common side effects were nausea, vomiting, and headache. These side effects are similar to what is commonly experienced with lower dosages. This study shows that LXB treatment can be adjusted for each person. Higher dosages (>9–12 g/night) may help some patients find further improvement in their narcolepsy symptoms.

**Graphical Abstract:**

**Figure:**
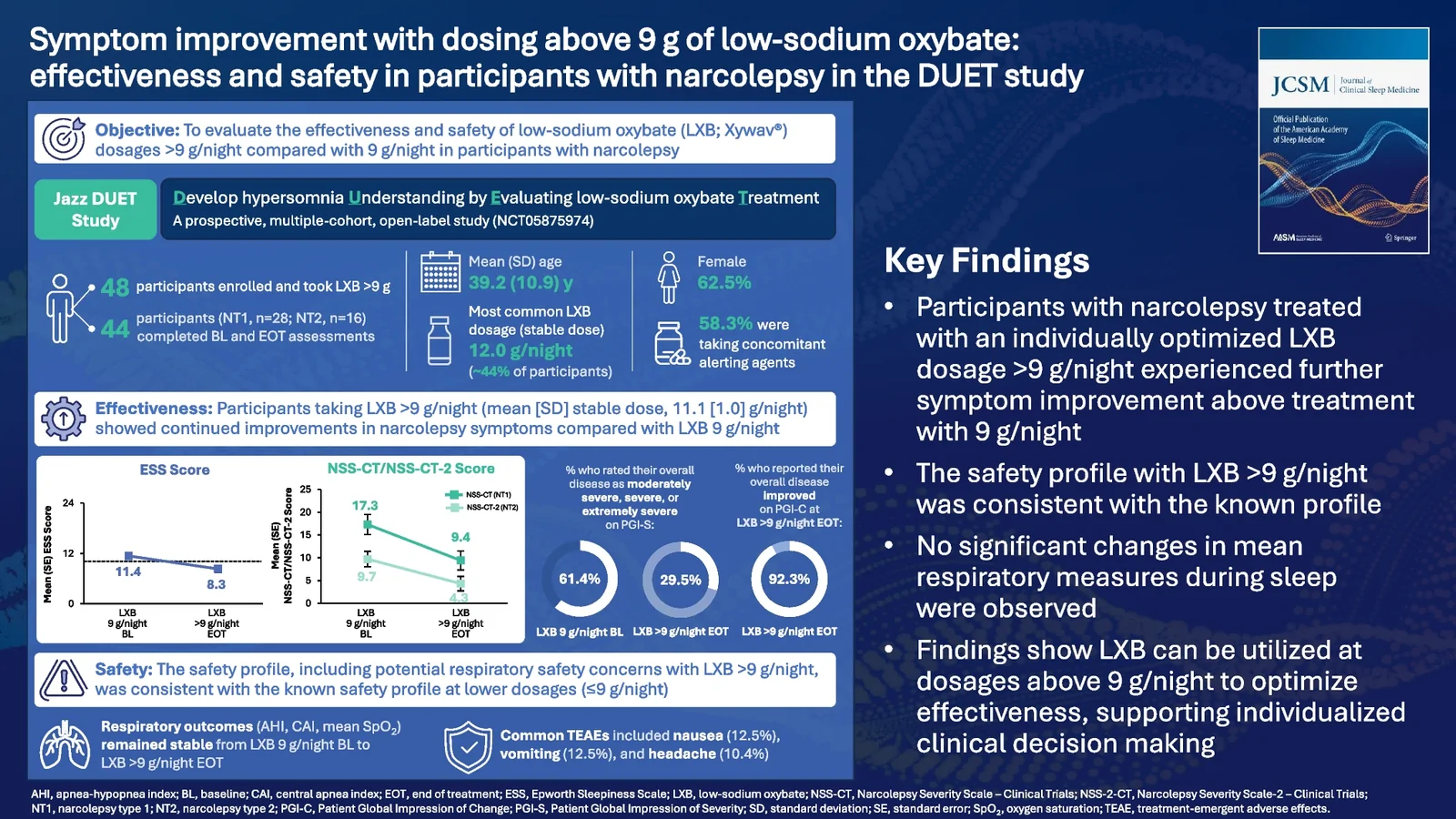

**Supplementary Information:**

The online version contains supplementary material available at https://doi.org/10.1007/s44470-026-00193-6.

## Introduction

Narcolepsy is a central disorder of hypersomnolence that is characterized by excessive daytime sleepiness (EDS), with other classic clinical features that include disrupted nighttime sleep (DNS), cataplexy, hypnagogic and hypnopompic hallucinations, and sleep paralysis [1]. All patients diagnosed with narcolepsy experience EDS and short rapid eye movement (REM) sleep onset latency (ie, increased tendency to transition into REM sleep following sleep onset), but other core symptoms may or may not be present. Narcolepsy comprises 2 subtypes that differ in symptom presentation [1]. Patients with narcolepsy type 1 (NT1) experience cataplexy and loss of hypothalamic hypocretin signalling (cerebrospinal fluid [CSF] hypocretin level ≤ 110 pg/mL). In narcolepsy type 2 (NT2), cataplexy is absent and CSF hypocretin is detectable. Narcolepsy can significantly impair daily functioning, work productivity, and quality of life, as well as increase socioeconomic burden [2–4].

Given the heterogeneity of symptom presentation, treatment needs also vary considerably. A recent qualitative interview study identified that patients with NT1 or NT2 can experience up to 30 different symptoms that may have up to 38 different functional impacts, highlighting the diversity of the lived experience of narcolepsy [5, 6]. In The 2025 update to quality measures for care of patients with narcolepsy, the American Academy of Sleep Medicine (AASM) noted that “treatment plans routinely change over time and can be affected by factors such as age, lifestyle, severity, tolerance, and comorbidities. Treatment choices should be tailored to each patient’s symptoms, comorbidities, tolerance, and risk of potential drug interaction [7], and treatment strategies that allow therapy to be individualized are needed for optimization of care.

Low-sodium oxybate (LXB; calcium, magnesium, potassium, and sodium oxybates; Xywav^®^), approved by the US Food and Drug Administration (FDA) for the treatment of cataplexy or EDS in individuals aged ≥ 7 years with narcolepsy and for the treatment of idiopathic hypersomnia in adults [8], allows for individualized dosing optimization. The recommended dosage range for adults with narcolepsy is 6–9 g/night, gradually titrated based on effectiveness and tolerability. The nightly dosage is divided into 2 equal or unequal doses (first dose at bedtime, second dose 2.5–4 h later). The maximum recommended dose, established in 2002 with the original FDA approval of sodium oxybate (SXB; Xyrem^®^; same active moiety as LXB), is 9 g/night [8, 9]; however, a retrospective analysis of adults enrolled in the Risk Evaluation and Mitigation Strategy (REMS) database found that approximately 1% of patients with narcolepsy were taking LXB dosages > 9 g/night (stable dose > 90 days) [10], and real-world data from the Nexus Narcolepsy Registry reported that 4.1% of patients taking SXB were taking dosages > 9 g/night [11]. To date, evidence supporting dosing regimens above 9 g/night has been limited. Clinical experience suggests higher dosages may be justified in select patients; therefore, it is critical to evaluate higher dosages to 1) ensure added benefit and 2) confirm no new safety concerns or worsening of known safety concerns.

The Jazz DUET (**D**evelop hypersomnia **U**nderstanding by **E**valuating low-sodium oxybate **T**reatment) study assessed the effectiveness and safety of LXB in treating sleep and daytime symptoms in people with narcolepsy or idiopathic hypersomnia. The patient-centered design of the DUET study allowed for individualized titration/dosing and continued use of stable concomitant alerting agents as needed for treatment optimization [12]. Primary findings for the narcolepsy and idiopathic hypersomnia cohorts have been published [13, 14]. Participants in the narcolepsy and idiopathic hypersomnia cohorts received LXB treatment according to the recommended dosage range (up to 9 g/night). Participants with narcolepsy who were currently taking LXB 9 g/night and who, in the opinion of the investigator, could benefit from higher dosages were enrolled in the greater than 9 g/night narcolepsy cohort (“ > 9 g cohort”) to explore dosage optimization up to 12 g/night. LXB dosages > 9 g/night were only evaluated in a separate cohort of participants with narcolepsy, and were not evaluated in participants with idiopathic hypersomnia. This manuscript evaluates the effectiveness and safety of using > 9–12 g/night compared with a 9 g/night baseline.

## Methods

### Study design

Jazz DUET was a prospective, multicenter, single-arm, multiple-cohort, open-label study (NCT05875974). Complete study methodology, including how input from a patient advisory board and clinician-scientist advisory board were integrated into the protocol to support a patient-centered approach, has been published [12]. Participants could enter the > 9 g cohort in 1 of 2 ways: 1) *Direct enrollment* – participants with narcolepsy taking prescription LXB 9 g/night as part of routine clinical care who, in the opinion of the investigator, would likely benefit from continued titration to dosages > 9 g/night, could enroll in the > 9 g cohort upon meeting screening criteria at study entry, or 2) *Transfer* – participants already enrolled in the narcolepsy cohort who had titrated up to 9 g/night and who, in the opinion of the investigator, could benefit from a higher LXB dosage, were discontinued from the narcolepsy cohort and enrolled in the > 9 g cohort.

**Fig. 1:**
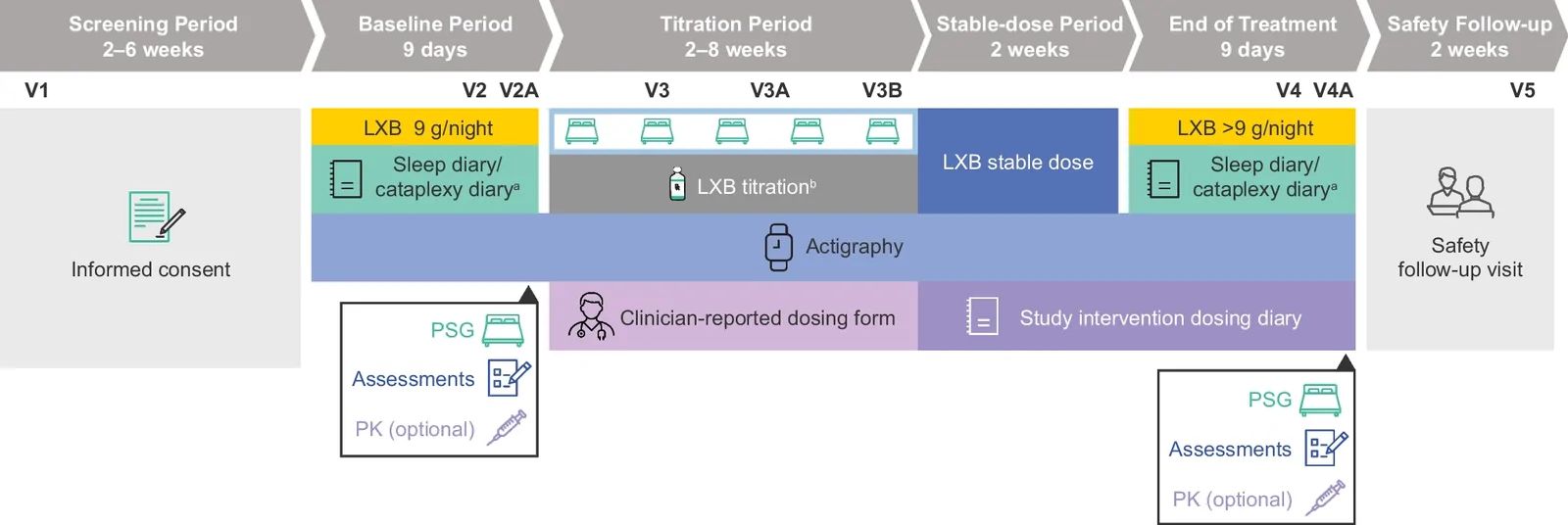
DUET study design for the > 9 g narcolepsy cohort. ^a^Cataplexy diary in narcolepsy type 1 only. ^b^Weekly titration visits with a dose escalation were performed as an overnight PSG visit for safety monitoring; visits without a dose escalation could be via teleconference. Titration could take between 2 and 8 weeks. V3 occurred on titration day 14. Additional in-clinic visits were scheduled for day 35 (V3A) and day 56 (V3B), as needed. Investigator could optimize participant dosage and move participant to SDP at V3, V3A, or V3B but not during intervening weekly teleconferences. *DUET*, Develop hypersomnia Understanding by Evaluating low-sodium oxybate Treatment; *LXB*, low-sodium oxybate; *PSG*, polysomnography; *SDP*, stable-dose period; *V*, visit

Study design for the > 9 g cohort included a screening period, 9-day baseline (BL) assessment on LXB 9 g (“LXB 9 g BL”), 2- to 8-week titration period, 2-week stable-dose period (SDP), 9-day end-of-treatment (EOT) period on optimized LXB > 9 g and ≤ 12 g (“optimized LXB > 9 g EOT”), and a safety follow-up visit approximately 2 weeks after the last study drug dosage (during safety follow-up, participants could return to commercial LXB as prescribed by their personal sleep physician) (Fig. 1). Total study duration was 11–22 weeks (11–36 weeks for transfer participants); range was dependent upon the duration of the screening and titration periods and whether the participant transferred from the narcolepsy cohort (which encompassed additional screening, baseline, and titration procedures).

The LXB 9 g BL period comprised 9 days of assessments (participants completed daily electronic diaries [eDiary] and wore actigraphy at home for 8 days) that ended with an overnight polysomnography (PSG) sleep clinic visit and an optional pharmacokinetic (PK) visit. Direct enrollment participants took their own commercial LXB during the assessment period and during the LXB 9 g BL PSG visit. After eligibility was confirmed at the LXB 9 g BL PSG visit, study intervention was dispensed. Transfer participants were provided LXB as study intervention after meeting initial eligibility criteria at the end of the narcolepsy cohort BL PSG visit.

During the titration period, participants took their first > 9 g/night dosage of study intervention (LXB 9.5 g/night) in the sleep clinic with PSG and safety monitoring. Each dosage escalation was limited to 0.5 g/night per week and was administered during an overnight PSG in the sleep clinic as a safety precaution and evaluation. Participants could titrate up to a maximum of LXB 12 g/night. All participants took LXB twice nightly, with the second dose taken approximately 4 h after the first dose. The total nightly dosage increase was focused on the first dose, which could be up to 7.5 g; the second dose could be up to 4.5 g. Within these limits, dosing adjustments were allowed per clinician judgement. Clinicians performed weekly titration check-ins to determine if the participant should increase, decrease, or maintain their dosage. While new dosage escalations resulted in a PSG clinic visit, weekly check-ins for a dosage that remained stable or decreased could be performed by telecommunication. Factors clinicians may have considered during optimization included maximizing effectiveness with improved symptom control (eg, EDS, cataplexy, waking refreshed) while managing adverse reactions [15]. Data on the clinician’s dosing decisions were captured with the clinician-reported dosing form.

Once the clinician determined the participant was taking an optimal dosage, this stable LXB dosage was maintained from the SDP through the optimized LXB > 9 g EOT period. Study procedures were the same for the LXB 9 g BL and optimized LXB > 9 g EOT periods (Fig. 1).

DUET was approved by relevant independent ethics committees or institutional review boards and was conducted in accordance with the Declaration of Helsinki. All participants provided written informed consent.

### Participants

Participants were males and females aged 18–75 years with a primary diagnosis of NT1 or NT2 according to *International Classification of Sleep Disorders*, third edition criteria [1] or *Diagnostic and Statistical Manual of Mental Disorders*, fifth edition criteria [16]. Participants must have been on a current LXB treatment regimen of 9 g/night (as part of routine clinical care or had reached LXB 9 g/night in the DUET narcolepsy cohort) and, per investigator’s judgment, be likely to benefit from taking a dosage > 9 g/night. Participants were allowed to continue concomitant non-oxybate anticataplectic or alerting agents (traditional stimulants or wake-promoting agents) and nicotine replacement therapy if the dosage was stable for ≥ 1 month before screening visit 1 with no plans to adjust the dosage during the study. Females were eligible if they were not pregnant or breastfeeding; if able to bear children, highly effective contraceptive (failure rate < 1%/year) was required. There was no Epworth Sleepiness Scale (ESS) score eligibility requirement at screening for the > 9 g cohort.

Key exclusion criteria included untreated or inadequately treated sleep-disordered breathing (SDB; apnea–hypopnea index [AHI] > 10 as assessed during the LXB 9 g BL PSG with hypopnea definition including a ≥ 4% desaturation as per Rule 1B of *The AASM Manual for the Scoring of Sleep and Associated Events* [17]); participants who had comorbid obstructive sleep apnea (OSA) were eligible if they were adequately treated for SDB (AHI ≤ 10); those participants were to maintain their SDB treatment regimen for the entirety of the study. Other sleep disorders, including parasomnias, circadian rhythm sleep disorders, or restless legs syndrome, were exclusionary. Participants taking medications known to have clinically significant central nervous system (CNS) sedating effects were excluded. Additional exclusion criteria included history or presence of any unstable or clinically significant medical (eg, chronic pain that may impact sleep), behavioral, psychiatric (including active suicidal ideation), neurologic, or surgical condition that may affect participant safety or interfere with study conduct.

### Outcomes

Key endpoints were changes from LXB 9 g BL to optimized LXB > 9 g EOT in ESS score, Narcolepsy Severity Scale – clinical trials version (NSS-CT and NSS-2-CT) total score and component scores (daytime sleepiness, cataplexy [NT1], hypnagogic and hypnopompic hallucinations, sleep paralysis, and DNS), frequency of weekly cataplexy attacks (NT1) from the NSS-CT and eDiary, and PSG-measured sleep parameters. Patient Global Impression of Severity (PGI-S) for overall disease was assessed at LXB 9 g BL and optimized LXB > 9 g EOT, and Patient Global Impression of Change (PGI-C) for overall disease was assessed at optimized LXB > 9 g EOT.

The ESS is an 8-item, patient-reported scale (total score: 0–24) that measures daytime sleepiness by assessing likelihood of “dozing” in various situations. Higher scores are associated with more severe daytime sleepiness; a total score > 10 indicates EDS [18]. A decrease of 2 points on the ESS is considered the clinical significance threshold [19].

The NSS-CT is a 15-item, patient-reported scale (total score: 0–57) that evaluates the severity, frequency, and impact of the pentad of narcolepsy symptoms (EDS, cataplexy, DNS, hypnagogic/hypnopompic hallucinations, and sleep paralysis). The CT version uses a 1-week lookback period. Higher NSS-CT total scores indicate more severe symptoms, with total scores 0–14 indicating mild, 15–28 moderate, 29–42 severe, and 43–57 very severe symptoms [20, 21]. The NSS-CT was administered to participants with NT1, whereas a modified version that omitted 3 questions (items 8–10) about cataplexy (total score: 0–44) was used for participants with NT2 (NSS-2-CT) [22]. Severity classification scores are not currently available for the NSS-2-CT; however, a longitudinal study estimated a minimal clinically important difference (MCID) of 3.35 points between untreated and treated patients [22].

Cataplexy attacks were assessed in participants with NT1 using the NSS-CT and eDiary. For NSS-CT, questions on the number of weekly cataplexy episodes (total, generalized, and partial) were assessed. Generalized cataplexy was defined as a loss of muscle tone all over, including collapse or inability to move. Partial cataplexy was defined as loss of muscle tone in only the face, neck, arms, or knees. For the eDiary, participants were provided with a definition of a cataplexy attack (“cause you to actually fall down or you would have fallen if you did not support yourself or make part of your body feel weak”) and instructed to record the number of daily cataplexy attacks experienced each night prior to bedtime. Weekly cataplexy attacks were calculated from daily assessments.

The PGI-S and PGI-C are 7-point, Likert-type scales that ask participants to rate their impression of the level of severity of their overall disease (ranging from 1 = “not present” to 7 = “extremely severe”) and the change in their overall disease (ranging from 1 = “very much improved” to 7 = “very much worse”), respectively.

The ESS, NSS-CT/NSS-2-CT, and PGI-S were completed (self-administered) during the LXB 9 g BL and optimized LXB > 9 g EOT PSG visits, and the PGI-C was completed (self-administered) during the optimized LXB > 9 g EOT PSG visit only. The eDiary was completed for 8 days prior to the LXB 9 g BL and again prior to the optimized LXB > 9 g EOT PSGs.

Safety endpoints included treatment-emergent adverse events (TEAEs), suicidal ideation/behaviors on the Columbia-Suicide Severity Rating Scale (C-SSRS), and sleep-related respiratory assessment measures. TEAEs were defined as any AE that began on or after the date of the first > 9 g/night dosage of study intervention until (and including) the date of the safety follow-up visit. TEAEs that occurred on the same date as (but prior to) titration to 9.5 g (ie, from the night before, after midnight) were captured as TEAEs. The C-SSRS was administered at every in-clinic visit and select telecommunication visits. Respiratory assessments included PSG-measured AHI, central apnea index (CAI), mean oxygen saturation (SpO_2_), and percentage of total sleep time (TST) spent with SpO_2_ < 90%. Respiratory assessments were measured at the LXB 9 g BL and optimized LXB > 9 g EOT PSG visits and were centrally scored.

### Statistical methods

The safety analysis set included all participants who enrolled in the study and took their prescribed LXB regimen for ≥ 1 night after the LXB 9 g BL period. The safety analysis set was used to summarize demographics and baseline characteristics, concomitant alerting medication use, and TEAEs. The completer analysis set included all participants who enrolled in the study, took their prescribed LXB regimen for ≥ 1 night after the LXB 9 g BL period, completed the SDP, and completed any assessments at the optimized LXB > 9 g EOT PSG visit. The completer analysis set was used to analyze all effectiveness outcomes and PSG-measured respiratory assessments. Data are presented for NT1 and NT2 combined (total > 9 g cohort) and separately. Demographic, concomitant medication use, drug exposure, and safety data are also presented separately for direct enrollment and transfer participants. Effectiveness outcomes were reported for participants who had both LXB 9 g BL and LXB > 9 g EOT assessments. Dosing during the SDP was reported for participants in the safety analysis set who completed the SDP.

Observed values and changes from LXB 9 g BL were summarized as continuous variables. Least squares mean (LSM) differences (SE; 95% confidence interval [CI]) were calculated using mixed model with repeated measures of change from LXB 9 g BL to optimized LXB > 9 g EOT, adjusted for the LXB 9 g BL value. One participant was considered an extreme outlier due to weekly cataplexy frequency values exceeding the upper bound for potential outliers, defined as the third quartile plus 1.5 times IQR at both LXB 9 g BL and LXB > 9 g EOT. This participant was removed from both NSS-derived and eDiary-derived cataplexy analyses. No formal hypothesis testing was planned for the > 9 g cohort.

## Results

### Participants

**Fig. 2:**
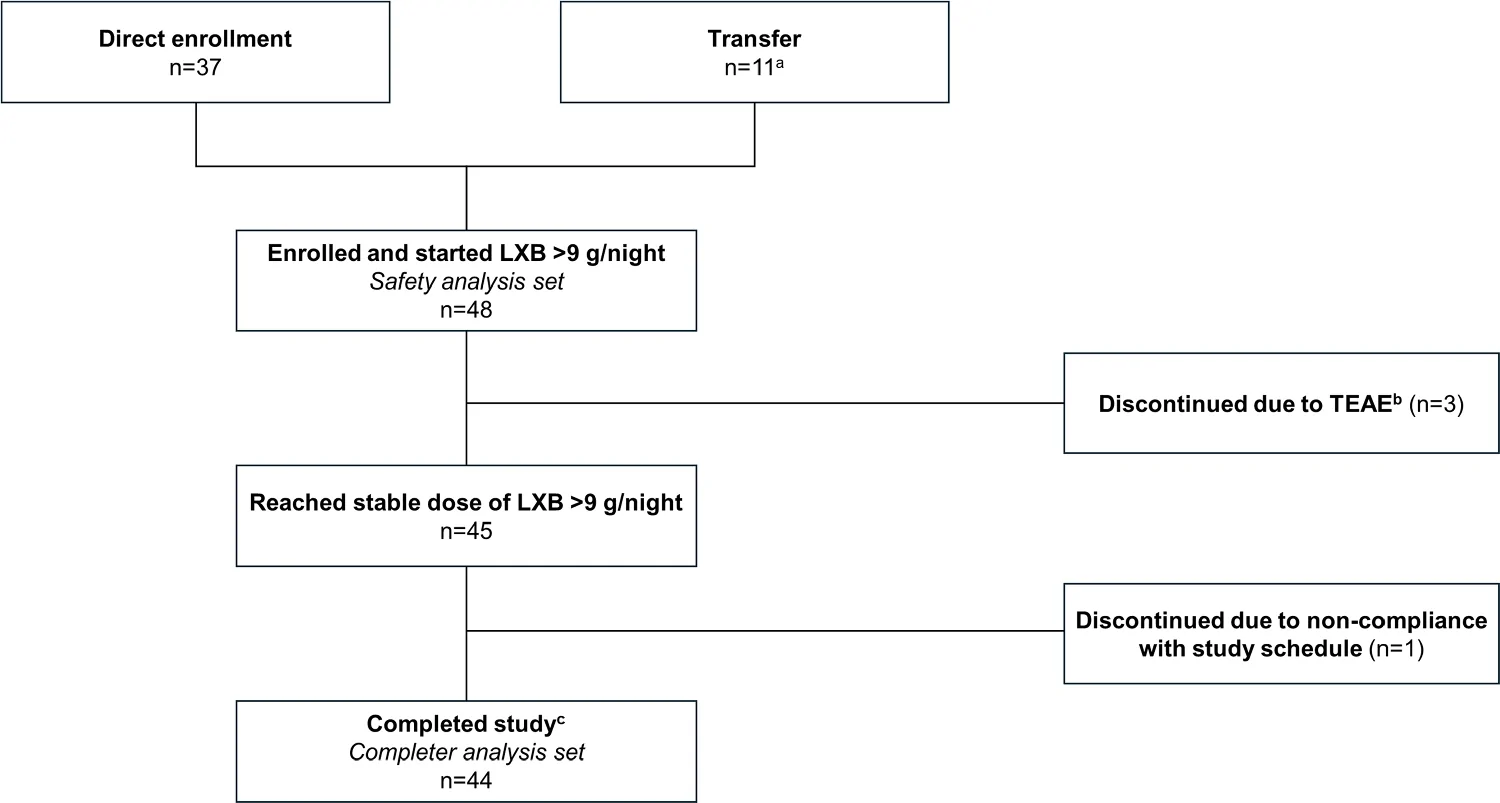
Participant disposition for the > 9 g narcolepsy cohort. ^a^A total of 13 participants transferred from the narcolepsy cohort to the > 9 g cohort; however, 2 transfer participants never took a dosage > 9 g (1 due to screening failure and 1 due to TEAEs of dysphoria and irritability). ^b^Three participants discontinued the study due to moderate TEAEs of nausea, anxiety, and fall, respectively. ^c^One participant had a TEAE of fall during the optimized LXB > 9 g EOT PSG visit; this participant completed the study, but the EOT PSG assessment was not completed. *EOT*, end of treatment; *LXB*, low-sodium oxybate; *PSG*, polysomnography; *TEAE*, treatment-emergent adverse event

A total of 48 (NT1, *n* = 30; NT2, *n* = 18) participants were included in the safety analysis set (Fig. 2). Of these, 37 (NT1, *n* = 22; NT2, *n* = 15) were enrolled directly from routine clinical care and 11 (NT1, *n* = 8; NT2, *n* = 3) transferred from the narcolepsy cohort. Forty-four (NT1, *n* = 28; NT2, *n* = 16) were included in the completer analysis set (Fig. 2).

Most participants were female (62.5%) and White (77.1%), with a mean (SD) age of 39.2 (10.9) years. Mean (SD) body mass index (BMI) was 29.9 (8.9) kg/m^2^, and 31.3% participants had OSA (all of whom were on positive airway pressure or oral appliance for the study duration) (Tables 1 and S1). All participants with OSA were direct enrollment participants.

**Table 1:** Demographics and baseline characteristics^a^

Characteristic	NT1 (*n* = 30)	NT2 (*n* = 18)	Total > 9 g narcolepsy cohort (*N* = 48)
Age, years			
Mean (SD)	38.2 (11.5)	40.8 (10.0)	39.2 (10.9)
Sex at birth, *n* (%)			
Male	10 (33.3)	8 (44.4)	18 (37.5)
Female	20 (66.7)	10 (55.6)	30 (62.5)
Gender identity, *n* (%)			
Male (including transgender man)	10 (33.3)	8 (44.4)	18 (37.5)
Female (including transgender woman)	20 (66.7)	10 (55.6)	30 (62.5)
Females of childbearing potential, *n* (%)^b^	16 (80.0)	7 (70.0)	23 (76.7)
Race, *n* (%)			
White	24 (80.0)	13 (72.2)	37 (77.1)
Black or African American	3 (10.0)	4 (22.2)	7 (14.6)
Asian	1 (3.3)	0	1 (2.1)
Multiple^c^	0	1 (5.6)	1 (2.1)
Ethnicity, *n* (%)			
Hispanic or Latino	1 (3.3)	0	1 (2.1)
Not Hispanic or Latino	29 (96.7)	18 (100)	47 (97.9)
Body mass index, kg/m^2^			
Mean (SD)	30.0 (8.0)	29.7 (10.5)	29.9 (8.9)
Comorbid obstructive sleep apnea,^d,e^*n* (%)	8 (26.7)	7 (38.9)	15 (31.3)

^a^Safety analysis set. ^b^Percentage calculated only for female sex at birth. ^c^Participant reported > 1 race. ^d^Per medical history and treatment records at screening and/or technical notes from any PSG visit where positive airway pressure treatment settings or oral appliances may have been mentioned. ^e^Of 15, 12 participants with comorbid obstructive sleep apnea were on sleep apnea treatment (PAP or oral appliance)

*NT1* narcolepsy type 1. *NT2* narcolepsy type 2. *PAP* continuous positive airway pressure. *PSG* polysomnography. *SD* standard deviation

At study entry, 58.3% of participants were taking concomitant alerting medications. The most commonly used alerting agents were amphetamines (31.3%) (Table S2).

### Dosing and treatment optimization

At screening for the > 9 g cohort, all participants were taking total nightly LXB dosages of 9 g/night. For direct enrollment participants, mean (SE) time on SXB prior to study entry was 34.5 (8.2) months (NT1, 23.4 [4.6]; NT2, 50.8 [18.6]). Median (Q1, Q3) time to titrate to a newly optimized stable dose was 55 (37, 68) days. During the SDP, mean (SD) total nightly LXB dosage was 11.1 (1.0) g/night (Table S3). A total LXB dosage of 12 g/night (7.5 g first dose, 4.5 g second dose) was reached by 21 participants (43.8%) (NT1, *n* = 13 [43.3%]; NT2, *n* = 8 [44.4%]). Thirteen unique dosing regimens were taken (Table S3).

### Excessive daytime sleepiness

**Fig. 3:**
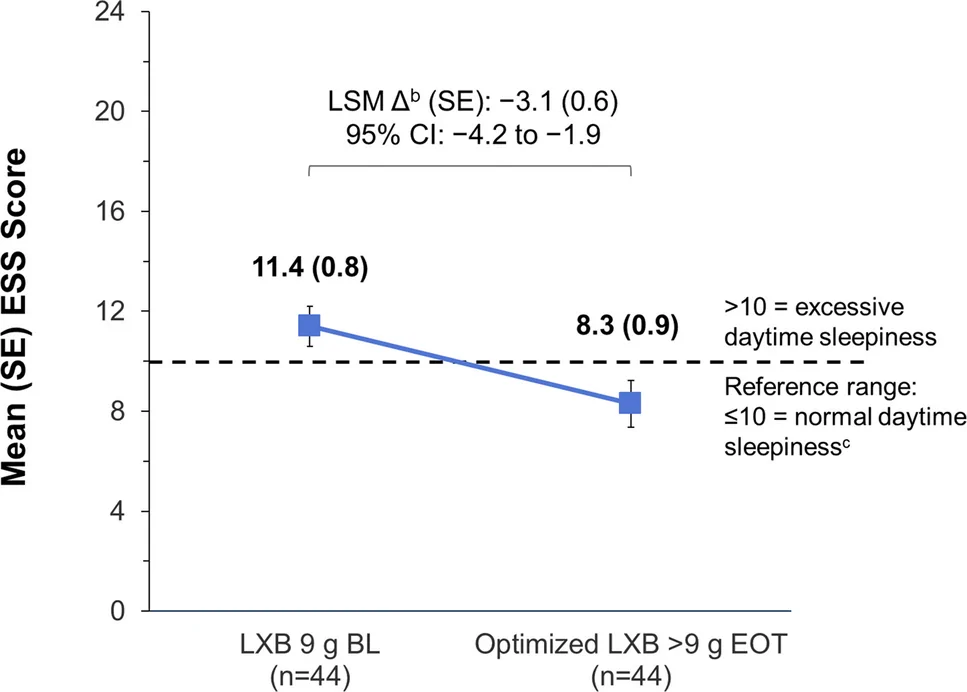
Epworth Sleepiness Scale.^a^ ^a^Completer analysis set. ^b^Difference between EOT and BL. ^c^Established categories consider scores up to 10 normal [18]. The MCID for ESS was defined as 2 points according to an AASM systematic review and meta-analysis [19]. *AASM*, American Academy of Sleep Medicine; *BL*, baseline; *CI*, confidence interval; *EOT*, end of treatment; *ESS*, Epworth Sleepiness Scale; *LSM*, least squares mean; *LXB*, low-sodium oxybate; *MCID*, minimal clinically important difference; *SE*, standard error

For the total > 9 g cohort (*n* = 44), mean (SE) ESS score was 11.4 (0.8) at LXB 9 g BL, which decreased to 8.3 (0.9) at optimized LXB > 9 g EOT (LSM difference: − 3.1; 95% CI: − 4.2, − 1.9) (Fig. 3). For participants with NT1 and NT2, mean (SE) ESS scores were 12.0 (1.1) and 10.3 (1.1) at LXB 9 g BL, which decreased to 9.2 (1.3) and 6.9 (1.3) at optimized LXB > 9 g EOT, respectively (NT1: LSM difference: − 2.8; 95% CI: − 4.3, − 1.4; NT2: LSM difference: − 3.5; 95% CI: − 5.4, − 1.6).

### Cataplexy

**Fig. 4:**
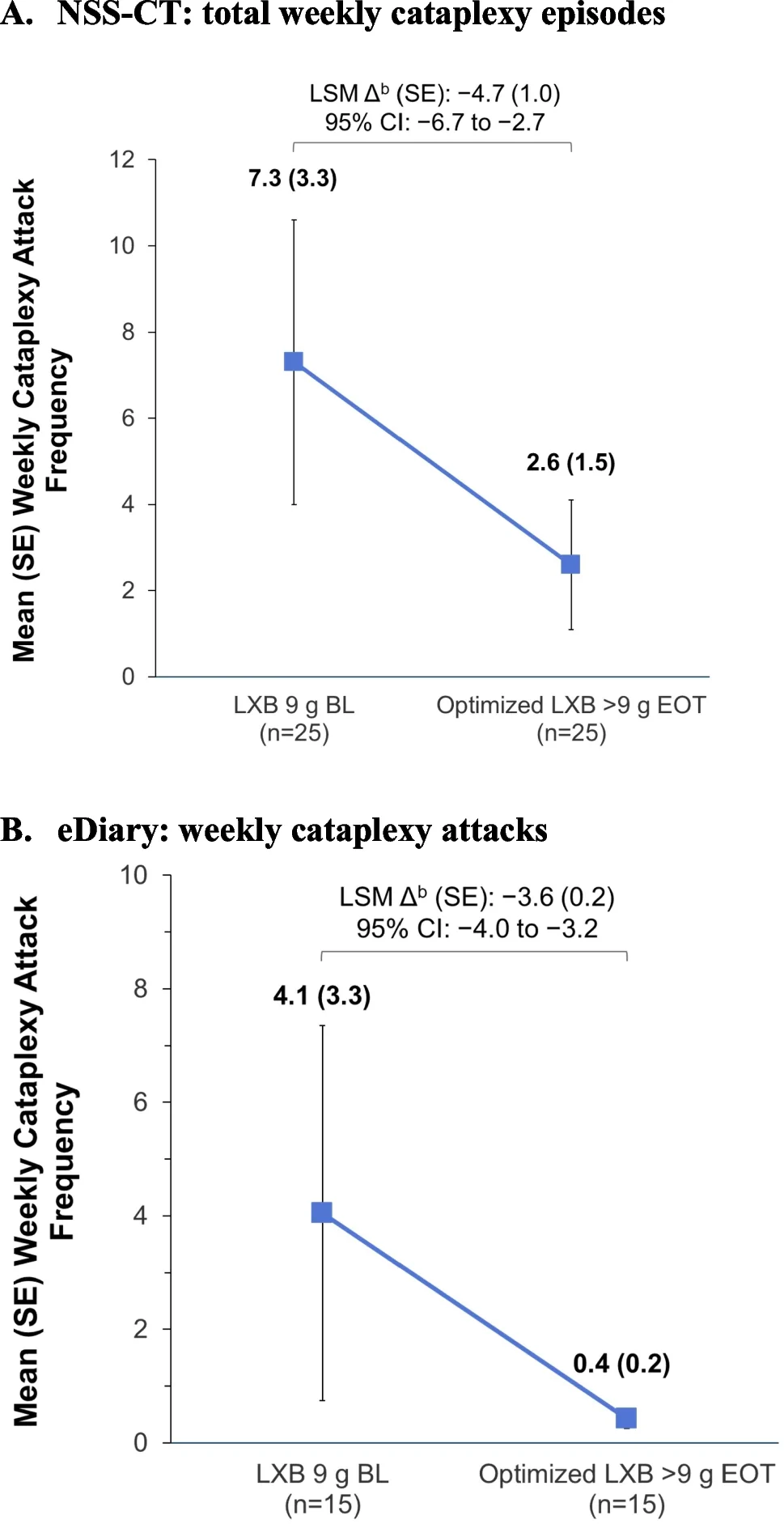
Weekly cataplexy frequency.^a^ ^a^Completer analysis set with 1 participant excluded from the analysis due to being an extreme outlier. ^b^Difference between EOT and BL. *BL*, baseline; *CI*, confidence interval; *EOT*, end of treatment; *LSM*, least squares mean; *LXB*, low-sodium oxybate; *NSS-CT*, Narcolepsy Severity Scale– clinical trial; *SE*, standard error

Based on the NSS-CT (*n* = 25), total number of weekly cataplexy episodes decreased from LXB 9 g BL (mean [SE]: 7.3 [3.3]) to optimized LXB > 9 g EOT (2.6 [1.5]) (LSM difference: − 4.7; 95% CI: − 6.7, − 2.7). Number of partial cataplexy episodes per week decreased from LXB 9 g BL (mean [SE]: 5.0 [2.2]) to optimized LXB > 9 g EOT (1.1 [0.6]) (LSM difference: − 3.8; 95% CI: − 4.8, − 2.9), and number of generalized episodes was similar (LXB 9 g BL: 2.4 [1.4]; optimized LXB > 9 g EOT: 1.5 [1.0]; LSM difference: − 0.8; 95% CI: − 1.8, 0.1) (Fig. 4A).

Based on the eDiary (*n* = 15), number of cataplexy attacks per week decreased from LXB 9 g BL (4.1 [3.3]) to optimized LXB > 9 g EOT (mean [SE]: 0.4 [0.2]) (LSM difference: − 3.6; 95% CI: − 4.0, − 3.2) (Fig. 4B).

### Narcolepsy symptom severity

**Fig. 5:**
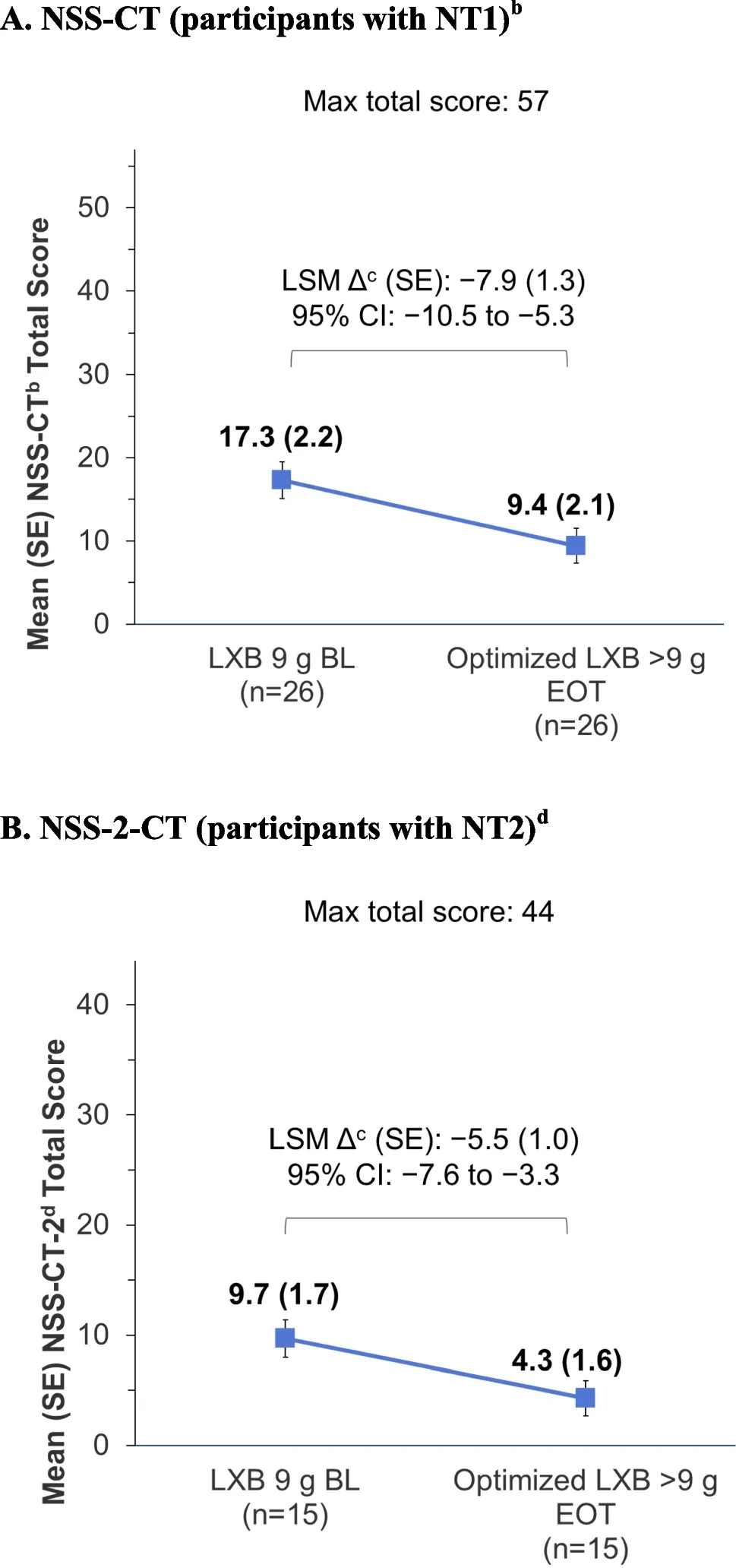
Narcolepsy Severity Scale – clinical trial total score.^a^ ^a^Completer analysis set. ^b^For the NSS-CT, levels of severity are as follows: very severe (43–57), severe (29–42), moderate (15–28), mild (0–14) [21].^c ^Difference between EOT and BL. ^d^For the NSS-CT-2, the MCID between untreated and treated patients was 3.35 with the Cohen effect size and 3.22 with the empirical rule effect size according to a longitudinal study (*n*= 26) [22]. *BL*, baseline; *CI*, confidence interval; *EOT*, end of treatment; *LSM*, least squares mean; *LXB*, low-sodium oxybate; *MCID*, minimal clinically important difference; *NSS-CT*, Narcolepsy Severity Scale – clinical trial; *NSS-2-CT*, Narcolepsy Severity Scale-2 – clinical trial; *NT1*, narcolepsy type 1; *NT2*, narcolepsy type 2; *SE*, standard error

For NT1, mean (SE) NSS-CT total score was 17.3 (2.2) at LXB 9 g BL (moderate severity [21]), which decreased to 9.4 (2.1) at optimized LXB > 9 g EOT (mild severity [21]) (LSM difference: − 7.9; 95% CI: − 10.5, − 5.3) (Fig. 5A). For NT2, mean (SE) NSS-2-CT total score was 9.7 (1.7) at LXB 9 g BL, which decreased to 4.3 (1.6) at optimized LXB > 9 g EOT (LSM difference: − 5.5; 95% CI: − 7.6, − 3.3) (Fig. 5B).

For both NT1 and NT2, mean (SE) NSS-CT and NSS-2-CT daytime sleepiness (NT1, 10.4 [1.1] to 5.6 [1.1]; NT2, 6.0 [1.1] to 2.3 [0.9]), cataplexy (NT1, 3.0 [0.7] to 1.6 [0.6]), hypnagogic/hypnopompic hallucinations (NT1, 1.3 [0.4] to 0.7 [0.3]; NT2, 1.1 [0.4] to 0.5 [0.4]), sleep paralysis (NT1, 0.9 [0.4] to 0.4 [0.2]; NT2, 0.7 [0.4] to 0.3 [0.2]), and DNS component scores (NT1, 1.8 [0.2] to 1.2 [0.2]; NT2, 1.9 [0.2] to 1.2 [0.2]) decreased (improved) from LXB 9 g BL to LXB > 9 g EOT (Figure S1).

### Overall disease: patient global impression assessments

**Fig. 6:**
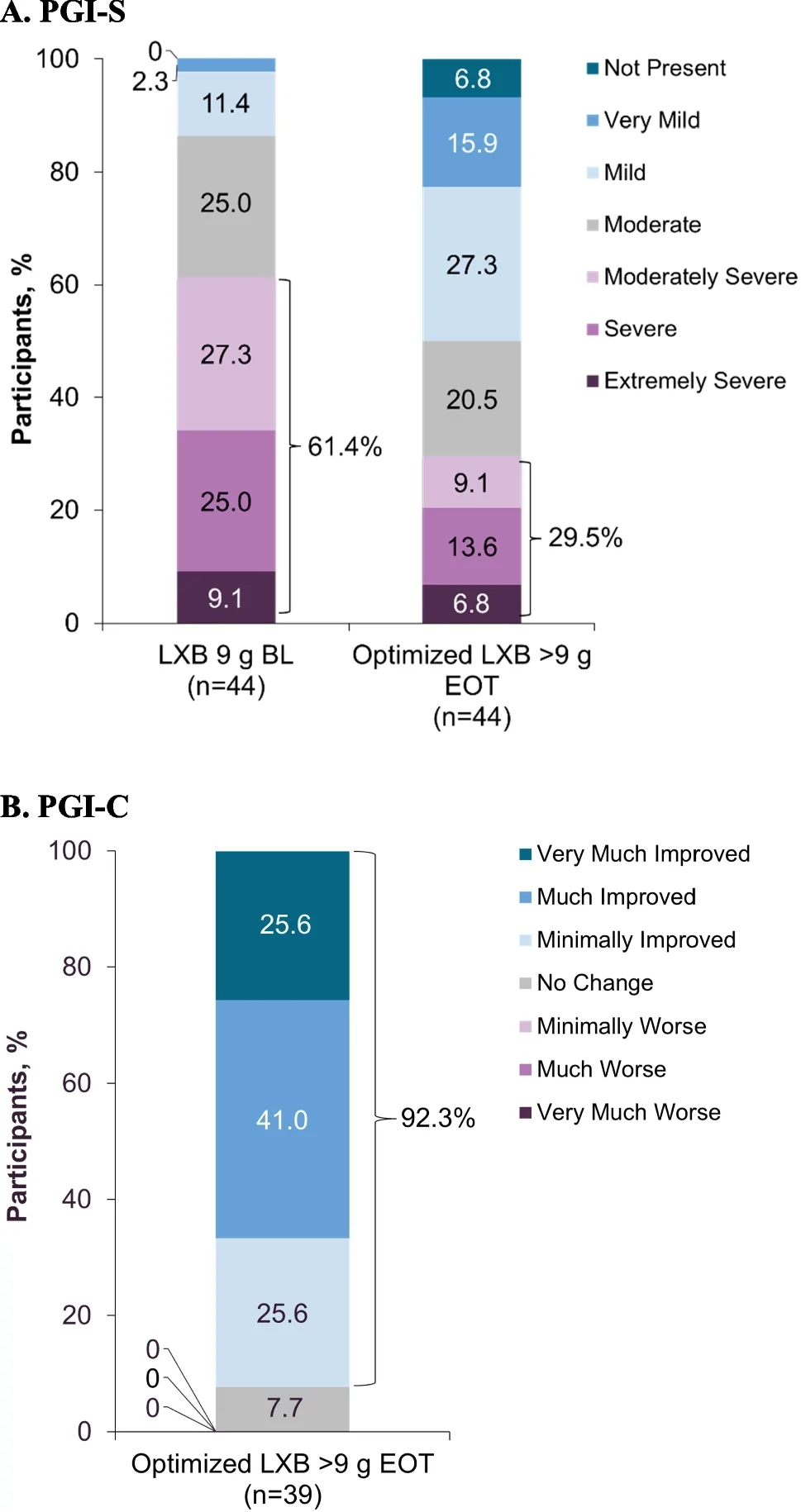
Patient Global Impression of Severity and Patient Global Impression of Change.^a^ ^a^Completer analysis set. *EOT*, end of treatment; *LXB*, low-sodium oxybate; *PGI-C*, Patient Global Impression of Change; *PGI-S*, Patient Global Impression of Severity

At LXB 9 g BL, 61.4% of participants in the total > 9 g cohort (NT1, 64.3%; NT2, 56.3%) rated their overall disease as severe (“moderately severe,” “severe,” or “extremely severe”) on the PGI-S (Fig. 6A). At optimized LXB > 9 g EOT, this percentage was 29.5% (NT1, 39.3%; NT2, 12.5%). On the PGI-C at optimized LXB > 9 g EOT, 92.3% of participants (NT1, 91.7%; NT2, 93.3%) reported that their overall disease improved (Fig. 6B). No participant reported that their disease worsened.

### PSG-measured sleep parameters

For the total > 9 g cohort, PSG-measured time in bed, total sleep time, sleep onset latency, REM latency, and sleep efficiency remained stable from LXB 9 g BL to LXB > 9 g EOT (Table S4).

### Safety

During titration (dosage escalation), mean (SD) number of safety PSGs conducted for each participant was 4.5 (1.7) (median, 5.0; min, 1; max, 6). There were no clinically meaningful changes in mean (SE) PSG-measured AHI, CAI, SpO_2_, or percentage of TST spent with SpO_2_ < 90% at optimized LXB > 9 g EOT compared with LXB 9 g BL (Table 2). All participants taking LXB dosages > 9 g/night who completed the EOT PSG (*n* = 43) had AHI ≤ 10 during the EOT PSG. Of these, 1 participant (with a medical history of OSA on continuous positive airway pressure [CPAP]) had elevated AHIs during 3 sequential LXB titration safety PSGs (AHI range, 16.5–22 events/h; predominantly central apneas); AHI was 1.5 events/h at the EOT PSG. Two participants who discontinued (neither of whom had a medical history of OSA) demonstrated elevated AHIs during LXB titration safety PSGs. One participant had elevated AHIs during 4 sequential LXB titration safety PSGs (AHI range, from 11.3 to 31.2 events/h; predominantly central apneas); AHI was 9.9 events/h at a subsequent titration safety PSG visit; this participant discontinued study intervention due to a TEAE of fall. The other participant had elevated AHIs during 3 sequential LXB titration safety PSGs (AHI range, from 11.6 to 22.9 events/h; predominantly obstructive apneas); this participant discontinued due to nonadherence with the study schedule. One participant experienced a single instance of narrow complex tachycardia during sleep during the first LXB titration safety PSG (maximum heart rate [HR], 158 bpm; average HR, 68 bpm); during all subsequent PSGs, including the EOT PSG, the participant’s maximum HR ranged from 80–90 bpm.

**Table 2:** Respiratory assessment measures (polysomnography)^a^

Characteristic	NT1 (*n* = 27)		NT2 (*n* = 16)		Total > 9 g narcolepsy cohort (*N* = 43)^b^	
	LXB 9 g BL	Optimized LXB > 9 g EOT	LXB 9 g BL	Optimized LXB > 9 g EOT	LXB 9 g BL	Optimized LXB > 9 g EOT
Apnea–hypopnea index (events/hour)						
Mean (SE)	2.0 (0.6)	1.6 (0.4)	1.3 (0.2)	1.6 (0.3)	1.8 (0.4)	1.6 (0.3)
LSM Δ^c^ (SE)	− 0.2 (0.4)		− 0.1 (0.5)		− 0.2 (0.3)	
95% CI	− 0.9, 0.5		− 1.0, 0.8		− 0.7, 0.4	
Central apnea index (events/hour)						
Mean (SE)	0.02 (0.01)	00.1 (0.01)	0.01 (0.00)	0.01 (0.00)	0.01 (0.01)	0.01 (0.00)
LSM Δ^c^ (SE)	0		0		0	
95% CI	(− 0.01, 0.01)		(− 0.01, 0.01)		(− 0.01, 0.01)	
Mean SpO_2_ (%)						
Mean (SE)	95.4 (0.3)	95.6 (0.2)	96.6 (0.3)	96.2 (0.2)	95.8 (0.2)	95.8 (0.1)
LSM Δ^c^ (SE)	− 0.1 (0.2)		0.1 (0.2)		0 (0.1)	
95% CI	− 0.4, 0.3		− 0.3, 0.5		− 0.2, 0.2	
Percentage of total sleep time spent with SpO_2_ < 90% (%)						
Mean (SE)	0.6 (0.3)	0.1 (0.1)	0.1 (0.1)	0.2 (0.2)	0.4 (0.2)	0.1 (0.1)
LSM Δ^c^ (SE)	− 0.3 (0.1)		− 0.1 (0.1)		− 0.3 (0.1)	
95% CI	− 0.5, − 0.2		− 0.4, 0.1		− 0.4, − 0.1	

^a^Completer analysis set. ^b^One participant had a TEAE of fall during the optimized LXB > 9 g EOT PSG visit; this participant completed the study, but the EOT PSG assessment was not completed. ^c^Difference between EOT and BL

*BL* baseline, *CI* confidence interval, *EOT* end of treatment, *LSM* least squares mean, *LXB* low-sodium oxybate, *NT1* narcolepsy type 1, *NT2* narcolepsy type 2, *PSG* polysomnography, *SE* standard error, *SpO*_*2*_ oxygen saturation, *TEAE* treatment-emergent adverse event

The most common TEAEs were nausea (12.5%), vomiting (12.5%), and headache (10.4%) (Table 3). Most were mild or moderate in severity. One serious TEAE of severe syncope (NT2) occurred during a morning blood draw for clinical labs after PSG; it was determined to be unrelated to LXB. Two severe non-serious TEAEs (NT1, hypnagogic hallucinations; NT2, nausea) occurred. Three TEAEs led to discontinuation (NT1, fall, anxiety; NT2, nausea) (Fig. 2). One participant with NT1 reported suicidal ideation on the C-SSRS during the titration period (day 35; depressive symptoms related to election), which resolved by the next visit; no suicidal ideation was reported for the rest of the study. TEAEs for direct enrollment and transfer participants are in Table S5.

**Table 3:** Treatment-emergent adverse events^a^

	NT1 (*n* = 30)	NT2 (*n* = 18)	Total > 9 g Narcolepsy Cohort (*N* = 48)
TEAEs, *n* (%)			
Participants with ≥ 1 TEAE	22 (73.3)	14 (77.8)	36 (75.0)
Treatment-related TEAE	17 (56.7)	9 (50.0)	26 (54.2)
Serious TEAE	0	1 (5.6)	1 (2.1)
TEAEs occurring in ≥ 5% of participants			
Nausea	2 (6.7)^b^	4 (22.2)^c^	6 (12.5)
Vomiting	3 (10.0)^b^	3 (16.7)^b^	6 (12.5)
Headache	3 (10.0)^c^	2 (11.1)^d^	5 (10.4)
Enuresis	4 (13.3)^e^	0	4 (8.3)
Hyperhidrosis	3 (10.0)^b^	1 (5.6)^d^	4 (8.3)
Somnolence	2 (6.7)^d^	2 (11.1)^b^	4 (8.3)
Diarrhea	2 (6.7)	1 (5.6)^d^	3 (6.3)
Fall	2 (6.7)^b^	1 (5.6)	3 (6.3)
Hypotension^f^	2 (6.7)^b^	1 (5.6)^d^	3 (6.3)
Pollakiuria	2 (6.7)^b^	1 (5.6)^d^	3 (6.3)
Upper respiratory tract infection	2 (6.7)	1 (5.6)	3 (6.3)

^a^Safety analysis set. ^b^Considered treatment-related, *n* = 2. ^c^Considered treatment-related, *n* = 3. ^d^Considered treatment-related, *n* = 1. ^e^Considered treatment-related, *n* = 4. ^f^The 3 hypotension TEAEs occurred during the overnight LXB 9 g BL pharmacokinetic visit while participants were taking LXB 9 g/night; all 3 TEAEs were moderate in severity, related to study intervention, and resolved; no associated changes in pulse, respiratory rate, or oxygen saturation were recorded in these participants

*NT1* narcolepsy type 1, *NT2* narcolepsy type 2, *TEAE* treatment-emergent adverse event

## Discussion

In the DUET study, participants with narcolepsy treated with an individually optimized LXB dosage > 9 g/night experienced further symptom benefit above treatment with 9 g/night, and TEAEs were comparable to those observed with lower dosages. Notably, respiratory parameters remained generally unchanged. This study fills a critical knowledge gap by providing evidence for the effectiveness and safety of LXB dosages > 9 g/night up to 12 g/night in participants identified by clinicians to potentially benefit from dosages higher than those recommended in the prescribing information.

When LXB was optimized to > 9 g/night, mean ESS scores improved by 3 points (decreasing from 11.4 to 8.3). This change is meaningful as it exceeds the AASM clinical significance threshold of 2 points [19]. Additionally, mean population scores improved to within the reference range for normal daytime sleepiness levels (≤ 10) [18]. These clinically meaningful improvements were met by both participants with NT1 and NT2. At baseline, participants continued to experience burdensome EDS (ESS scores > 10) [18], despite taking LXB 9 g/night. Additionally, 58% of participants were concurrently taking alerting agents. Notably, there was no minimum baseline ESS score inclusion criterion for the > 9 g cohort. EOT ESS scores for participants in the > 9 g cohort (mean [SE], 8.3 [0.9]) were similar to EOT ESS scores for participants in the narcolepsy cohort taking LXB up to 9 g/night (mean [SE], 8.6 [1.0]), suggesting some participants may require higher dosages to achieve similar benefit in EDS [13].

When LXB was increased from 9 g/night to > 9 g/night, NSS-CT- and eDiary-based mean number of weekly cataplexy episodes decreased by 36% (from 7.3 to 2.6) and 10% (from 4.1 to 0.4), respectively, demonstrating that participants with NT1 could experience further improvement in cataplexy symptoms with higher dosages. The AASM clinical significance threshold is 25% [19].

In addition to optimizing EDS and cataplexy, higher LXB dosages were associated with further improvements in overall symptom/disease burden. NSS-CT total scores indicated that some participants with NT1 continued to experience moderate symptoms when taking LXB 9 g/night [20, 21]. When LXB was increased, narcolepsy symptoms improved to mild [22]. For participants with NT2, NSS-2-CT total scores decreased by 5.5 points, exceeding the MCID threshold of 3.4 [22]. NSS-CT and NSS-2-CT component scores revealed that daytime sleepiness, cataplexy (NT1 only), hypnagogic/hypnopompic hallucinations, sleep paralysis, and DNS all improved when LXB was increased to > 9 g/night in participants with NT1 and NT2. These data suggest that some participants continued to experience bothersome symptoms across the pentad despite treatment with LXB 9 g/night, and symptoms were able to improve with higher dosages. Mean EOT NSS-CT and NSS-2-CT scores in participants taking higher LXB dosages were similar to mean EOT scores in the narcolepsy cohort taking up to 9 g/night, further supporting that some participants needed higher dosages for similar symptom abatement [13]. Accordingly, after treatment with higher LXB dosages, the percentage of participants reporting their overall disease as “severe” or worse (on the PGI-S) substantially reduced for both participants with NT1 and NT2. Together, these results indicate that higher dosages of LXB improved overall symptoms and disease severity.

Oxybate (SXB or LXB) dosages have typically not exceeded 9 g/night in clinical studies. Early small studies used weight-based dosages (50–60 mg/kg [23–25], which would be equivalent to 3.5–4.2 g for a 70 kg individual), and a PK study used 6 g/night (2 equal doses of 3 g) [10, 11]. The first use of a 9 g dosage was in an SXB phase 3 registration study (dosage cohorts of 3, 6, and 9 g/night). Results showed a strong dose-dependent reduction in cataplexy of up to 69% with the 9 g dosage [26]. Subsequent studies retained 9 g/night as the highest dosage; however, recent data from a narcolepsy registry and separate data from the REMS program revealed that 4% and 1% of participants, respectively, were taking dosages > 9 g/night, warranting investigation into > 9 g/night dosages.

A key clinical concern with use of higher LXB dosages is the potential for increased safety risk, particularly for SDB. As a CNS depressant, the LXB prescribing label contains a warning that respiratory depression can occur and to use caution when considering the use of LXB with other CNS depressants (which were exclusion criteria in DUET) [8]. In the current study, no significant changes in AHI, CAI, SpO_2_, or central apnea index were observed from LXB 9 g BL to optimized LXB > 9 g EOT. To prioritize safety, participants with AHI ≥ 10 were excluded, and participants with OSA (AHI < 10) were asked to maintain stable use of their SDB treatment throughout the study. Results indicate that stable use of SDB treatment continued to adequately control OSA severity during treatment with higher LXB dosages. While these data highlight no significant changes in respiratory parameters for patients with minimal or adequately treated SDB, it is unknown whether more severe OSA or other forms of SDB (eg, central sleep apnea, nocturnal hypoventilation) would have a similar response. A crossover study (George et al.) in patients with OSA who were not on CPAP found that SXB 9 g/night did not significantly increase AHI or SpO_2_; however, central apnea frequency significantly increased, and significant oxygen desaturations were observed in 3 participants [27]. In another trial, lower dosages of SXB (4.5 g/night) did not increase AHI, number of obstructive or central apneic events, or SpO_2_ [28]. These data highlight the importance of monitoring for emergence of or changes in SDB with LXB or SXB treatment, and the importance of continued adherence with SDB treatment in patients with OSA. Additionally, physicians should carefully evaluate concomitant medication use (restricting other CNS depressants).

The overall safety profile of LXB > 9 g/night, including the most common TEAEs and overall TEAE rate, was consistent with the known safety profile at lower dosages for both NT1 and NT2 [13, 29]. Nausea, vomiting, and headache were the most common TEAEs for LXB > 9 g/night. Although sample sizes for the > 9 g cohort were small, data suggest that, compared with recommended dosages of LXB, no new or worsening adverse effects were observed with higher dosages.

Results of this study should not be generalized to other oxybate products, as formulations differ in dosing, administration schedules, and sodium content. LXB contains 92% less sodium than high-sodium oxybate formulations. A 9-g dosage of LXB contains 131 mg of sodium [30], roughly 6% of the recommended maximum daily sodium limit of 2300 mg/day [31–34]. In contrast, a 9-g dosage of high-sodium oxybate contains 1640 mg of sodium [9], about 71% of that limit and exceeding the AHA-recommended ideal limit of 1500 mg/day [33]. At higher dosages (up to 12 g), sodium exposure from high-sodium oxybate can reach 2200 mg—approximately 96% of the recommended maximum daily amount—which may increase risk of sodium-related cardiovascular/cardiometabolic complications. The cardiovascular concerns of high-sodium oxybate have been published [35, 36], and this higher sodium exposure would amplify such concerns.

The decision to invite patients to enroll in the > 9 g cohort was up to the clinician’s judgment with respect to whether the participant would benefit from additional symptom improvement. Some differences were seen in transfer participants compared with their narcolepsy cohort peers, with transfer participants exhibiting higher BMI (mean [SD] BMI, 32.6 [8.3] vs 29.5 [6.7] kg/m^2^) and greater concomitant alerting agent use (64% vs 56%), particularly amphetamine aspartate/amphetamine sulfate and methylphenidate [13]. The greater use of stimulants may point to a more severe narcolepsy disease in these participants. Further research is needed to understand whether patient populations most likely to benefit from higher dosages have distinguishing clinical features other than remaining symptoms after treatment with LXB 9 g/night. These data suggest that patients with remaining symptoms may warrant consideration of an escalation in therapy.

This is the first clinical study to evaluate the effectiveness and safety of LXB dosages > 9 g/night, and this report provides evidence supporting individualized treatment optimization to further improve narcolepsy symptoms with higher dosages. A key strength of the DUET study is the protocol allowed for individualized dosing. Clinicians utilized over a dozen unique dosing regimens during individual treatment optimization, demonstrating the flexibility of LXB dosing to meet individual patient needs. Additionally, the DUET study used a patient-centric design to prioritize participant wellbeing [12].

Despite the study’s strengths, there were several limitations. The open-label design and lack of a control group limit the ability to causally attribute findings to LXB. Participants with sleep apnea were asked to maintain stable use of their SDB treatment throughout the study; however, adherence was not objectively monitored or recorded. LXB dosing was limited to twice-nightly regimens where the first dose could not exceed 7.5 g and the second dose could not exceed 4.5 g, limiting understanding of LXB efficacy in other dosing regimen options (eg, thrice-nightly dosing). Effectiveness outcomes were evaluated only in participants who reached LXB > 9 g EOT assessments. Finally, additional research is needed to elucidate clinical features of patient populations best suited for continued titration to higher dosages and to understand the significance of optimizing treatment.

This evaluation of the effectiveness and safety of LXB dosages > 9 g/night demonstrates that some patients with narcolepsy may experience further therapeutic benefit in overall disease improvement with higher dosages (up to 12 g/night). Data show that the safety profile, including respiratory parameters, is similar to the known safety profile at lower dosages. This study supports that LXB dosing can be increased to optimize effectiveness and tolerability for individual patients.

## Supplementary Information

Below is the link to the electronic supplementary material.

ESM 1 (DOCX 390 KB)

## Data Availability

All relevant data are provided within the manuscript and supporting files. Jazz has established a process to review requests from qualified external researchers for data from Jazz-sponsored clinical trials in a responsible manner that includes protecting patient privacy, assurance of data security and integrity, and furthering scientific and medical innovation. Additional details on Jazz Pharmaceuticals data sharing criteria and process for requesting access can be found at: https://www.jazzpharma.com/science/clinical-trial-data-sharing/.

## Abbreviations

AASM: American Academy of Sleep Medicine
AHI: Apnea-hypopnea index
BL: Baseline
BMI: Body mass index
CAI: Central apnea index
CI: Confidence interval
CPAP: Continuous positive airway pressure
CNS: Central nervous system
CSF: Cerebral spinal fluid
C-SSRS: Columbia-Suicide Severity Rating Scale
DNS: Disrupted nighttime sleep
DUET: Develop hypersomnia Understanding by Evaluating low-sodium oxybate Treatment
eDiary: Electronic diary
EDS: Excessive daytime sleepiness
EOT: End of treatment
ESS: Epworth Sleepiness Scale
FDA: US Food and Drug Administration
HR: Heart rate
LSM: Least squares mean
LXB: Low-sodium oxybate
LXB 9 g BL: 9-Day baseline on LXB 9 g/night
MCID: Minimal clinically important difference
NSS-CT: Narcolepsy Severity Scale–clinical trials version
NT1: Narcolepsy type 1
NT2: Narcolepsy type 2
Optimized LXB > 9 g EOT: 9-Day end-of-treatment on LXB > 9 g– ≤ 12 g
OSA: Obstructive sleep apnea
PAP: Positive airway pressure
PGI-C: Patient Global Impression of Change
PGI-S: Patient Global Impression of Severity
PK: Pharmacokinetic
PSG: Polysomnography
REM: Rapid eye movement
REMS: Risk Evaluation and Mitigation Strategy
SD: Standard deviation
SDB: Sleep-disordered breathing
SDP: Stable dose period
SE: Standard error
SpO_2_: Mean oxygen saturation
SXB: Sodium oxybate
TEAE: Treatment-emergent adverse event
TST: Total sleep time
