# Supplementary material for: Symptom improvement with dosing above 9 g of low-sodium oxybate: effectiveness and safety in participants with narcolepsy in the DUET study
Source: J Clin Sleep Med. 2026 Oct 15;22(1):198. doi: 10.1007/s44470-026-00193-6 (PMC13672759; doi:10.1007/s44470-026-00193-6)
Supplement: Supplementary file 1 [file 44470_2026_193_MOESM1_ESM.docx]

**Symptom Improvement With Dosing Above 9 Grams of Low-Sodium Oxybate: Effectiveness and Safety in Participants With Narcolepsy in the DUET Study**

Jerald H. Simmons, MD^1,2^; Logan D. Schneider, MD^3^; Chad M. Ruoff, MD^4^; David T. Plante, MD, PhD^5^; Deborah A. Nichols, MS^6^; Teresa L. Steininger, PhD^6^; Marisa Whalen, PharmD^7^; M. Todd Kirby, PhD^7,8^; Jing Dai, PhD^6^; Alyssa Cairns, PhD^7^; Richard K. Bogan, MD^9^

^1^Comprehensive Sleep Medicine Associates, Houston, TX, USA; ^2^Sleep Education Consortium, Sugar Land, TX, USA; ^3^Stanford University Center for Sleep Sciences and Medicine, Stanford, CA, USA; ^4^Mayo Clinic, Scottsdale, AZ, USA; ^5^Department of Psychiatry, University of Wisconsin–Madison, Madison, WI, USA; ^6^Jazz Pharmaceuticals, Palo Alto, CA, USA; ^7^Jazz Pharmaceuticals, Philadelphia, PA, USA; ^8^Department of Psychiatry & Health Behavior, Medical College of Georgia at Augusta University, Augusta, GA, USA; ^9^University of South Carolina School of Medicine, Columbia, SC, USA

**SUPPLEMENTAL MATERIAL**

**Table S1 Demographics and baseline characteristics in direct enrollment and transfer participants.^a^**

|  | **Direct enrollment** | | | **Transfer** | | |
| --- | --- | --- | --- | --- | --- | --- |
| **Characteristic** | **NT1**  **(n=22)** | **NT2**  **(n=15)** | **Total (n=37)** | **NT1**  **(n=8)** | **NT2**  **(n=3)** | **Total**  **(n=11)** |
| **Age, years** |  |  |  |  |  |  |
| Mean (SD) | 39.8 (11.8) | 43.3 (8.3) | 41.2 (10.5) | 33.9 (10.3) | 28.0 (8.0) | 32.3 (9.7) |
| **Sex at birth, n (%)** |  |  |  |  |  |  |
| Male | 7 (31.8) | 8 (53.3) | 15 (40.5) | 3 (37.5) | 0 | 3 (27.3) |
| Female | 15 (68.2) | 7 (46.7) | 22 (59.5) | 5 (62.5) | 3 (100) | 8 (72.7) |
| **Gender identity, n (%)** |  |  |  |  |  |  |
| Male (including transgender man) | 7 (31.8) | 8 (53.3) | 15 (40.5) | 3 (37.5) | 0 | 3 (27.3) |
| Female (including transgender woman) | 15 (68.2) | 7 (46.7) | 22 (59.5) | 5 (62.5) | 3 (100) | 8 (72.7) |
| **Participant of childbearing potential, n (%)^b^** | 11 (73.3) | 4 (57.1) | 15 (68.2) | 5 (100) | 3 (100) | 8 (100) |
| **Race, n (%)^c^** |  |  |  |  |  |  |
| White | 16 (72.7) | 12 (80.0) | 28 (75.7) | 8 (100) | 1 (33.3) | 9 (81.8) |
| Black or African American | 3 (13.6) | 3 (20.0) | 6 (16.2) | 0 | 1 (33.3) | 1 (9.1) |
| Asian | 1 (4.5) | 0 | 1 (2.7) | 0 | 0 | 0 |
| Multiple^d^ | 0 | 0 | 0 | 0 | 1 (33.3) | 1 (9.1) |
| **Ethnicity, n (%)** |  |  |  |  |  |  |
| Not Hispanic or Latino | 21 (95.5) | 15 (100) | 36 (97.3) | 8 (100) | 3 (100) | 11 (100) |
| **Body mass index, kg/m^2^** |  |  |  |  |  |  |
| Mean (SD) | 28.8 (7.9) | 29.6 (10.8) | 29.1 (9.0) | 33.4 (7.8) | 30.7 (11.2) | 32.6 (8.3) |
| **Comorbid obstructive sleep apnea,^e^ n (%)** | 8 (36.4) | 7 (46.7) | 15 (40.5) | 0 | 0 | 0 |

^a^Safety analysis set. ^b^Percentage calculated only for female sex at birth. ^c^Race for 2 participants (direct enrollment) was unknown. ^d^Participant reported >1 race. ^e^Per medical history and treatment records at screening and/or technical notes from any PSG visit where positive airway pressure treatment settings or oral appliances may have been mentioned

PSG, polysomnography; SD, standard deviation; V, visit.

**Table S2 Concomitant alerting agent medications.^a,b^**

| **Total >9 g narcolepsy cohort** | | | |
| --- | --- | --- | --- |
| **ATC Level 4 Term, n (%)**  **Preferred Term, n (%)** | **NT1**  **(n=30)** | **NT2**  **(n=18)** | **Total**  **(N=48)** |
| **Participants taking a concomitant alerting agent,^c,d^ n (%)** | 16 (53.3) | 12 (66.7) | 28 (58.3) |
| **Centrally acting sympathomimetics** | 14 (46.7) | 10 (55.6) | 24 (50.0) |
| Amphetamine aspartate, amphetamine sulfate, dexamphetamine saccharate, dexamphetamine sulfate | 9 (30.0) | 6 (33.3) | 15 (31.3) |
| Solriamfetol hydrochloride | 2 (6.7) | 2 (11.1) | 4 (8.3) |
| Methylphenidate | 3 (10.0) | 1 (5.6) | 4 (8.3) |
| Armodafinil | 1 (3.3) | 1 (5.6) | 2 (4.2) |
| Lisdexamphetamine mesilate | 1 (3.3) | 1 (5.6) | 2 (4.2) |
| Amphetamine sulfate | 0 | 1 (5.6) | 1 (2.1) |
| Bupropion hydrochloride | 0 | 1 (5.6) | 1 (2.1) |
| **Other nervous system drugs** | 5 (16.7) | 3 (16.7) | 8 (16.7) |
| Pitolisant hydrochloride | 5 (16.7) | 3 (16.7) | 8 (16.7) |
| **Direct enrollment** | | | |
| **ATC Level 4 Term, n (%)**  **Preferred Term, n (%)** | **NT1**  **(n=22)** | **NT2**  **(n=15)** | **Total**  **(n=37)** |
| **Participants taking a concomitant alerting agent,^c,d^ n (%)** | 11 (50.0) | 10 (66.7) | 21 (56.8) |
| **Centrally acting sympathomimetics** | 9 (40.9) | 8 (53.3) | 17 (45.9) |
| Amphetamine aspartate, amphetamine sulfate, dexamphetamine saccharate, dexamphetamine sulfate | 6 (27.3) | 5 (33.3) | 17 (45.9) |
| Solriamfetol hydrochloride | 1 (4.5) | 2 (13.3) | 3 (8.1) |
| Methylphenidate | 2 (9.1) | 0 | 2 (5.4) |
| Armodafinil | 0 | 1 (6.7) | 1 (2.7) |
| Lisdexamphetamine mesilate | 1 (4.5) | 1 (6.7) | 1 (2.7) |
| Amfetamine sulfate | 0 | 1 (6.7) | 1 (2.7) |
| Bupropion hydrochloride | 0 | 1 (6.7) | 1 (2.7) |
| **Other nervous system drugs** |  |  |  |
| Pitolisant hydrochloride | 4 (18.2) | 3 (20.0) | 7 (18.9) |
| **Transfer** | | | |
| **ATC Level 4 Term, n (%)**  **Preferred Term, n (%)** | **NT1**  **(n=8)** | **NT2**  **(n=3)** | **Total**  **(n=11)** |
| **Participants taking a concomitant alerting agent,^c,d^ n (%)** | 5 (62.5) | 2 (66.7) | 7 (63.6) |
| **Centrally acting sympathomimetics** |  |  |  |
| Amphetamine aspartate, amphetamine sulfate | 3 (37.5) | 1 (33.3) | 4 (36.4) |
| Solriamfetol hydrochloride | 1 (12.5) | 0 | 1 (9.1) |
| Methylphenidate | 1 (12.5) | 1 (33.3) | 2 (18.2) |
| Armodafinil | 1 (12.5) | 0 | 1 (9.1) |
| **Other nervous system drugs** |  |  |  |
| Pitolisant hydrochloride | 1 (12.5) | 0 | 1 (9.1) |

^a^Safety analysis set. ^b^Participants could have been taking multiple different alerting medications. ^c^It is not known whether these agents were prescribed for excessive sleepiness, narcolepsy, idiopathic hypersomnia, or another condition. ^d^Concomitant medications were started prior to the first dose of LXB and were ongoing throughout the study.

ATC, anatomical therapeutic chemical; NT1, narcolepsy type 1; NT2, narcolepsy type 2.

**Table S3 Mean nightly LXB dosage during stable-dose period.^a,b^**

| **LXB Dosage, g** | **NT1**  **(n=28)** | **NT2**  **(n=17)** | **Total >9 g Narcolepsy Cohort**  **(N=45^b^)** |
| --- | --- | --- | --- |
| **Overall total nightly LXB dosage** |  |  |  |
| Mean (SD) | 11.1 (1.0) | 11.1 (1.0) | 11.1 (1.0) |
| Median (min, max) | 11.5 (9.0, 12.0) | 11.5 (9.0, 12.0) | 11.5 (9.0, 12.0) |
| **Twice-nightly LXB dosage^c^** |  |  |  |
| First nightly LXB dose |  |  |  |
| Mean (SD) | 6.6 (1.1) | 6.6 (1.1) | 6.6 (1.1) |
| Median (min, max) | 7.0 (4.5, 7.5) | 7.0 (4.5, 7.5) | 7.0 (4.5, 7.5) |
| Second nightly LXB dose |  |  |  |
| Mean (SD) | 4.4 (0.2) | 4.5 (0.1) | 4.5 (0.2) |
| Median (min, max) | 4.5 (3.5, 4.8) | 4.5 (4.5, 5.0) | 4.5 (3.5, 5.0) |
| **Most common dosing regimens (first/second),^d^ n (%)** |  |  |  |
| 7.5 g / 4.5 g | 13 (43.3) | 8 (44.4) | 21 (43.8) |
| 6.0 g / 4.5 g | 3 (10.0) | 2 (11.1) | 5 (10.4) |
| 7.0 g / 4.5 g | 2 (6.7) | 1 (5.6) | 3 (6.3) |
| 4.5 g / 4.5 g | 1 (3.3) | 2 (11.1) | 3 (6.3) |
| 6.5 g / 4.5 g | 1 (3.3) | 1 (5.6) | 2 (4.2) |
| 6.5 g / 4.0 g | 2 (6.7) | 0 | 2 (4.2) |
| 5.5 g / 4.5 g | 1 (3.3) | 1 (5.6) | 2 (4.2) |
| 5.0 g / 4.5 g | 2 (6.7) | 0 | 2 (4.2) |
| 7.0 g / 3.5 g | 1 (3.3) | 0 | 1 (2.1) |
| 5.5 g / 5.0 g | 0 | 1 (5.6) | 1 (2.1) |
| 5.25 g / 4.5 g | 0 | 1 (5.6) | 1 (2.1) |
| 4.75 g / 4.75 g | 1 (3.3) | 0 | 1 (2.1) |
| 4.5 g / 4.75 g | 1 (3.3) | 0 | 1 (2.1) |

^a^Includes all participants from the safety analysis set who reached the SDP. ^b^Once a participant reached an optimized dosage based on investigator judgment, they continued this dosage as a stable regimen during the SDP and EOT periods. One participant downtitrated to 9.0 g/night and later withdrew due to an AE of nausea. ^c^All participants took LXB twice nightly. ^d^In ≥5% of participants in total narcolepsy cohort.

AE, adverse event; EOT, end of treatment; LXB, low-sodium oxybate; max, maximum; min, minimum; NT1, narcolepsy type 1; NT2, narcolepsy type 2; SD, standard deviation; SDP, stable-dose period.

**Table S4 PSG-measured sleep parameters**

| **Parameter** | **LXB 9 g BL,**  **mean (SE)**  **(n=44)** | **Optimized LXB >9 g (EOT),**  **mean (SE)**  **(n=44)** | **LS mean Δ^b^**  **(95% CI)** |
| --- | --- | --- | --- |
| **Time in bed (min)** | 494.0 (10.2) | 509.9 (9.9) | 15.9 (–2.9, 34.7) |
| **Total sleep time (min)** | 392.3 (12.4) | 411.9 (12.6) | 19.6 (–0.4, 39.5) |
| **Sleep onset latency (min)** | 18.5 (2.9) | 18.0 (2.8) | –0.5 (–5.4, 4.3) |
| **REM latency (min)** | 171.8 (19.1) | 173.8 (21.7) | 2.0 (–39.9, 44.0) |
| **Sleep efficiency (%)** | 79.5 (1.9) | 80.7 (1.9) | 1.2 (–1.3, 3.7) |

^a^Completer analysis set. ^b^Difference between EOT and BL.

CI, confidence interval; EOT, end of treatment; LS, least squares; LXB, low-sodium oxybate; min, minute; PSG, polysomnography; REM, rapid eye movement; SE, standard error; TIB, time in bed.

**Table S5 Treatment-emergent adverse events in direct enrollment and transfer participants.^a^**

|  | **Direct Enrollment** | | | **Transfer^b^** | | |
| --- | --- | --- | --- | --- | --- | --- |
| **TEAEs, n (%)** | **NT1**  **(n=22)** | **NT2**  **(n=15)** | **Total**  **(n=37)** | **NT1**  **(n=8)** | **NT2**  **(n=3)** | **Total (n=11)** |
| Participants with ≥1 TEAE | 15 (68.2) | 11 (73.3) | 26 (70.3) | 7 (87.5) | 3 (100) | 10 (90.9) |
| Treatment-related TEAE | 12 (54.5) | 7 (46.7) | 19 (51.4) | 5 (62.5) | 2 (66.7) | 7 (63.6) |
| TEAEs occurring in ≥5% of participants in the total >9 g narcolepsy cohort | | | | | | |
| Nausea | 1 (4.5) | 2 (13.3) | 3 (8.1) | 1 (12.5) | 2 (66.7) | 3 (27.3) |
| Vomiting | 1 (4.5) | 1 (6.7) | 2 (5.4) | 2 (25.0) | 2 (66.7) | 4 (36.4) |
| Headache | 2 (9.1) | 2 (13.3) | 4 (10.8) | 1 (12.5) | 0 | 1 (9.1) |
| Enuresis | 1 (4.5) | 0 | 1 (2.7) | 3 (37.5) | 0 | 3 (27.3) |
| Hyperhidrosis | 2 (9.2) | 1 (6.7) | 3 (8.1) | 1 (12.5) | 0 | 1 (9.1) |
| Somnolence | 1 (4.5) | 2 (13.3) | 3 (8.1) | 1 (12.5) | 0 | 1 (9.1) |
| Diarrhea | 0 | 1 (6.7) | 1 (2.7) | 2 (25.0) | 0 | 2 (18.2) |
| Fall | 1 (4.5) | 1 (6.7) | 2 (5.4) | 1 (12.5) | 0 | 1 (9.1) |
| Hypotension | 0 | 0 | 0 | 2 (25.0) | 1 (33.3) | 3 (27.3) |
| Pollakiuria | 1 (4.5) | 0 | 1 (2.7) | 1 (12.5) | 1 (33.3) | 2 (18.2) |
| Upper respiratory tract infection | 0 | 1 (6.7) | 1 (2.7) | 2 (25.0) | 0 | 2 (18.2) |

^a^Safety analysis set. ^b^A TEAE is defined as an AE that began on or after the date of the first >9 g dose of study intervention until (and including) the date of the safety follow-up visit.

AE, adverse event; NT1, narcolepsy type 1; NT2, narcolepsy type 2; TEAE, treatment-emergent adverse event.

**Figure S1 Narcolepsy Severity Scale – Clinical Trial Component Scores^a^ Stratified by NT1 and NT2**

A. **Daytime Sleepiness^b^**

B. **Cataplexy^d^**

C. **Hypnagogic/Hypnopompic Hallucinations^e^**

D. **Sleep Paralysis^f^**

E. **DNS^g^**

^a^Completer analysis set. ^b^Includes items related to daytime sleepiness (items 1–7) on the NSS-CT/NSS-2-CT. ^c^Difference between EOT and BL. ^d^Includes items related to frequency and impact of cataplexy episodes (items 8–10) on the NSS-CT.  ^e^Includes items related to hypnagogic/hypnopompic hallucinations (items 11–12 on the NSS-CT and items 8–9 on the NSS-2-CT). ^f^Includes items related to sleep paralysis (items 13–14 on the NSS-CT and items 10–11 on the NSS-2-CT). ^g^Includes items related to DNS (item 15 on the NSS-CT and item 12 on the NSS-2-CT).

BL, baseline; CI, confidence interval; DNS, disrupted nighttime sleep; EOT, end of treatment; LSM, least squares mean; LXB, low-sodium oxybate; NSS-CT, Narcolepsy Severity Scale – clinical trial; NSS-2-CT, Narcolepsy Severity Scale-2 – clinical trial; NT1, narcolepsy type 1; NT2, narcolepsy type 2; SE, standard error.
